# SU6668 suppresses proliferation of triple negative breast cancer cells through down-regulating MTDH expression

**DOI:** 10.1186/1475-2867-13-88

**Published:** 2013-08-29

**Authors:** Lu Wang, Zhaozhe Liu, Dongchu Ma, Ying Piao, Fang Guo, Yaling Han, Xiaodong Xie

**Affiliations:** 1 Oncology Department, Cancer Treatment Center, General Hospital of Shenyang Military Region, Shenyang, P. R. China; 2 Medical Experimental Department, Cancer Treatment Center, General Hospital of Shenyang Military Region, Shenyang, P. R. China; 3 Department of Cardiology, Institute of Cardiovascular Research of People’s Liberation Army, General Hospital of Shenyang Military Region, Shenyang, P. R. China

**Keywords:** SU6668, Triple- negative breast cancer, Polyploidization, MTDH

## Abstract

**Background:**

The multiple tyrosine kinase inhibitors SU6668 have a promising therapeutic effect on the progression of hematological malignancies and some solid tumors. Here, we determined its effect on triple negative breast cancer (TNBC) cells and explored the potential molecular mechanism.

**Methods:**

In this study, MDA-MB-231 cells were treated with SU6668 (15 μM, 30 μM) for 72 h and the change of proliferation was examined by MTT and tablet cloning. DNA ploidy was detected by flow cytometric analysis with PI staining. Double-label immunofluorescence method was used to detect the expression and distribution of MTDH proteins. VEGFR2, HIF-1α, MTDH, E-cadhrein, and SMA expressions were detected by Western bolt assay.

**Results:**

This study showed that SU6668 inhibited the proliferation and induced polyploidization of MDA-MB-231 cells in a dose dependent form. SU6668 exposure increased the distribution of MTDH in cytoplasm and decreased its distribution in nuclei. After the treatment of SU6668, VEGFR2, HIF-1α, MTDH and SMA proteins were down-regulated, while E-cadhrein was up-regulated in MDA-MB-231 cells.

**Conclusions:**

In conclusion, SU6668 exposure maybe induces polyploidization, inhibit EMT and influence the expression of MTDH, which suppresses the proliferation in TNBC cells. MTDH is a key signal protein in downstream of VEGF/HIF-1αpathway in MDA-MB-231 cells, which may be used as the potential target in the treatment of TNBC.

## Background

TNBC is defined as estrogen receptor, progesterone receptor and human epidermal growth factor receptor type 2 (HER2) negativity. Its incidence accounts for 15% of breast cancers. Although the proportion is not high, it is easy to recurrence and metastasis with a poor prognosis of malignant characteristics [1,2]. Due to the lack of endocrine and HER2-target therapeutics, the treatment for TNBC is still the hot spot and difficulty of present clinical research. So far, there is no special guideline for TNBC treatment. Because of its chemo sensitivity, most of clinicians choose anthracycline-based and yew-based chemotherapy regimens. Some studies focus on microtubules stabilizer (e.g. Docetaxel) and receptor tyrosine kinase inhibitor (e.g. Erlotinib), which can suppress tumor proliferation by blocking mitosis and receptor tyrosine kinase [3].

MTDH gene, also called astrocyte elevated gene 1 (AEG-1) gene, was originally from human embryonic astrocytes cloning. It is significantly over-expressed in many tumor tissues and closely related to proliferation, angiogenesis, invasion, metastasis and treatment resistance of breast cancer; it has the potential to be an effective therapy target gene [4,5].

Polyploidy plays an important role in development of cells, and is also related to human diseases, especially cancer [6]. Although polyploidy occur frequently in most multicellular organisms and human cancers, but the knowledge of polyploidy state function is still quite limited. A polyploid cell can be either blocked in the cell cycle, or regain potentially cancerous proliferation. Recent studies suggested that some drug could induce Polyploidization in breast cancer cells, and effect its anti-tumor activity by blocking mitosis, inhibiting cell proliferation ability and promote the cell-aging process [7].

Excessive activation of tyrosine kinase is closely related to tumorigenesis, development, prognosis and outcome [8]. More and more tyrosine kinase inhibitors has been developed to be new antitumor drugs. As one of them, SU6668 was discovered in 2000 [9]. Studies showed that it inhibited receptor tyrosine phosphorylation and mitosis to suppress tumor cell proliferation, and had a therapeutic effect on a variety of solid tumors and hematological malignancies [10-12]. However, the effect of SU6668 on TNBC has not yet been reported.

## Results

### SU6668 suppressed proliferation in MDA-MB-231 cells

**Figure 1:**
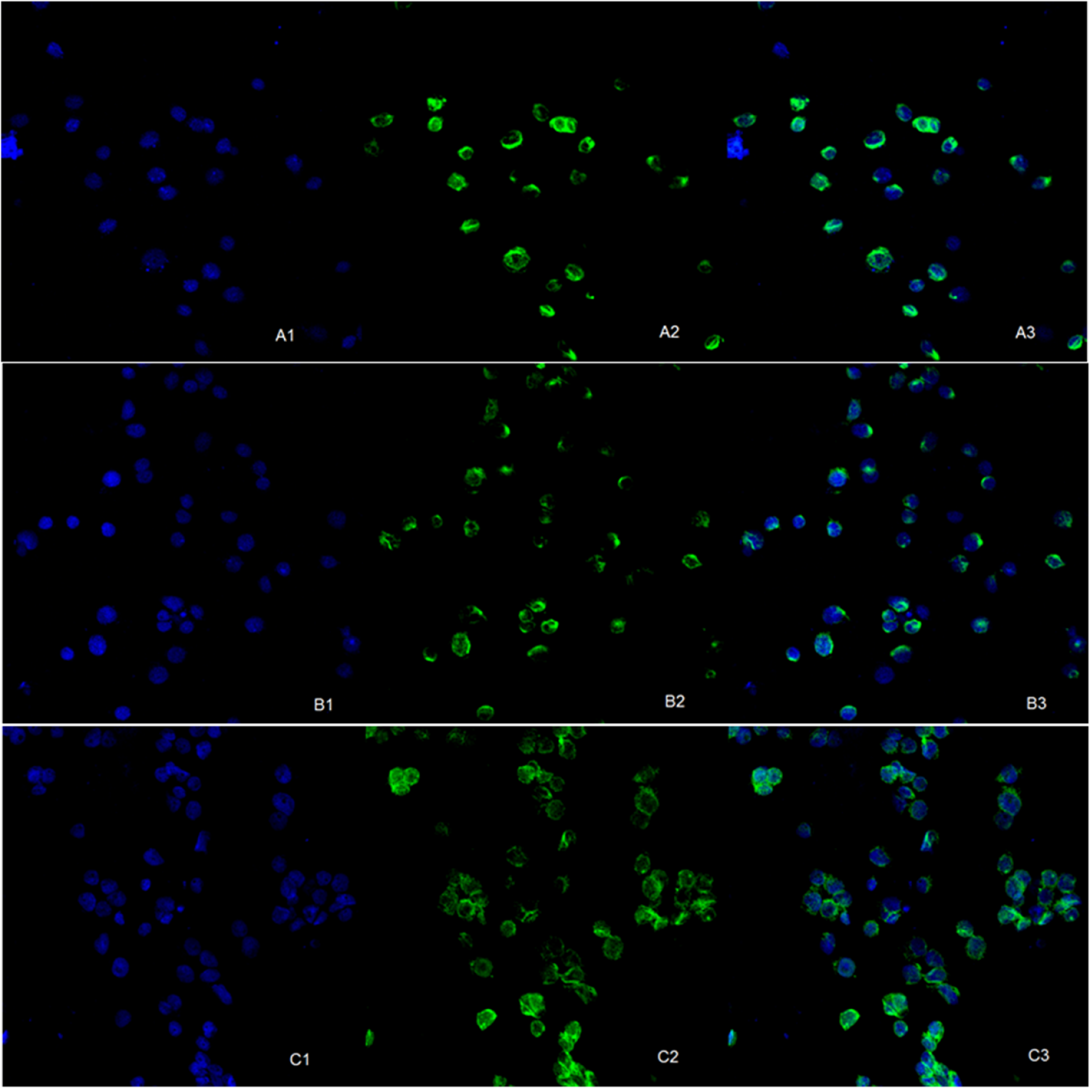
**Concentration-suppression curve of MDA-MB-231 cells treated with SU6668.** MDA-MB-231 cells were cultured in media supplemented with SU6668 (0-60μM) for 72h. The media were then replaced with MTT working reagent for 4 h, and DMSO was added finally.

Treated with different concentrations SU6668, MDA-MB-231 cells proliferation was significantly inhibited in a dose dependence form. In the concentration of 15 μM and 30 μM, the cell inhibitory rate were 10% and 20% respectively, so 15 μM and 30 μM were chosen for our later experiments (Table 1, Figure 1).

**Table 1:** Absorbance value and cell growth inhibition rate of MDA-MB-231 cells treated with different concentrations SU6668

**Concentration of SU(μM)**	**n**	**OD value**	**Growth inhibition rate (%)**
0	5	1.74 ± 0.26	-
6.25	5	1.68 ± 0.17	4.55 ± 0.98
12.5	5	1.63 ± 0.08	6.32 ± 1.01
18.75	5	1.54 ± 0.13	11.49 ± 0.53
25	5	1.46 ± 0.06	16.09 ± 1.17
31.25	5	1.27 ± 0.11	27.01 ± 0.82
37.5	5	0.97 ± 0.08	44.25 ± 1.36
43.75	5	0.82 ± 0.09	52.87 ± 2.01
50	5	0.47 ± 0.12	72.99 ± 1.94

### SU6668 inhibited clone formation in MDA-MB-231 cells

**Figure 2:**
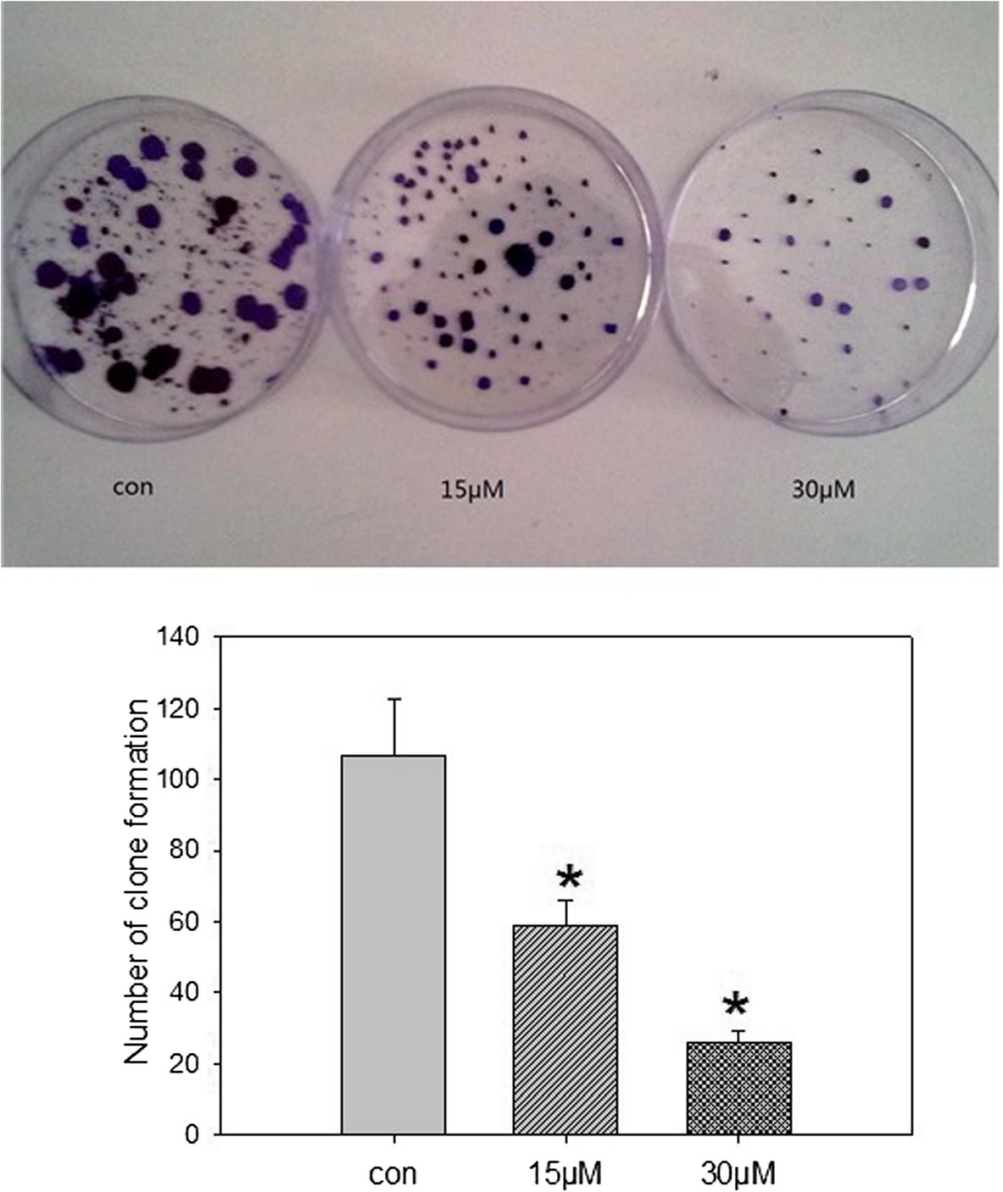
**Clone formation of MDA-MB-231 cells treated with SU6668.** MDA-MB-231 cells were cultured in media supplemented with SU6668(15μM, 30μM) for 72h. After three weeks, cells were fixed with methanol for 15 min, then stained with Giemsa for 20 min. ∗means compared with control group, *P*<0.05.

After SU6668 treatment, the single colony of 15 μM and 30 μM group was obviously smaller than control grouped, the number of colony decreased significantly (59 ±7.1, 26 ± 3.5) than the control group (107 ± 15.4) in a dose dependence form (Figure 2).

### SU6668 influenced the morphology in MDA-MB-231 cells

**Figure 3:**
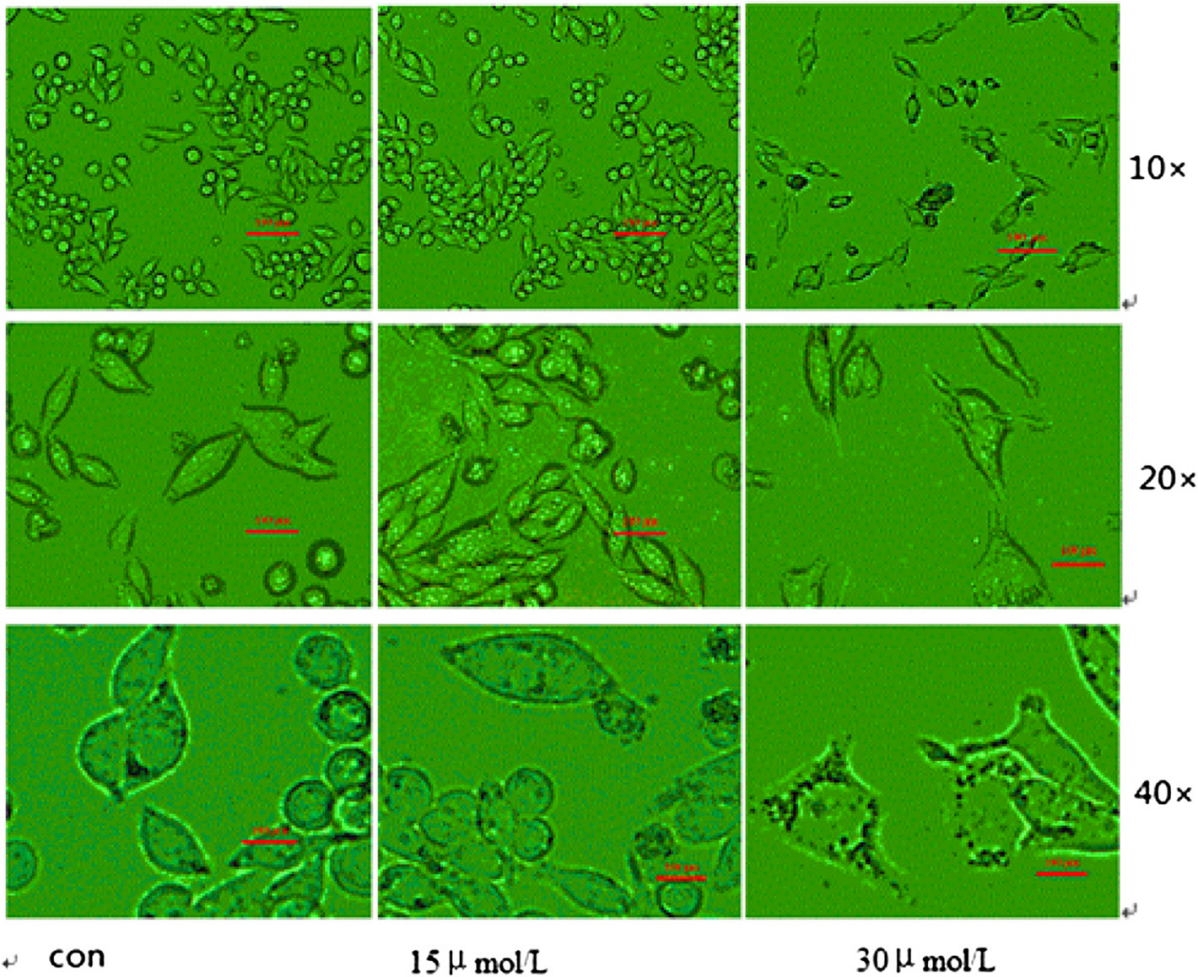
**Morphologic change of MDA-MB-231 cells treated with SU6668.** MDA-MB-231 cells were cultured in media supplemented with SU6668(15μM, 30μM) for 72h, and observed under optical microscopy with 10, 20 and 40-fold magnification.

Under optical microscopy, we observed that cells’ contour was clear and connected closely in 15 μM group, while cells’ morphology had no significant change in the control group. In 30 μM group, the cells’ contour was fuzzy, transparency was reduced, intercellular structure was loose. Meanwhile, the number of nuclei was increased and a small amount of apoptosis morphological changes was found at high magnification (Figure 3).

### SU6668 induced DNA polyploidization in MDA-MB-231 cells

**Figure 4:**
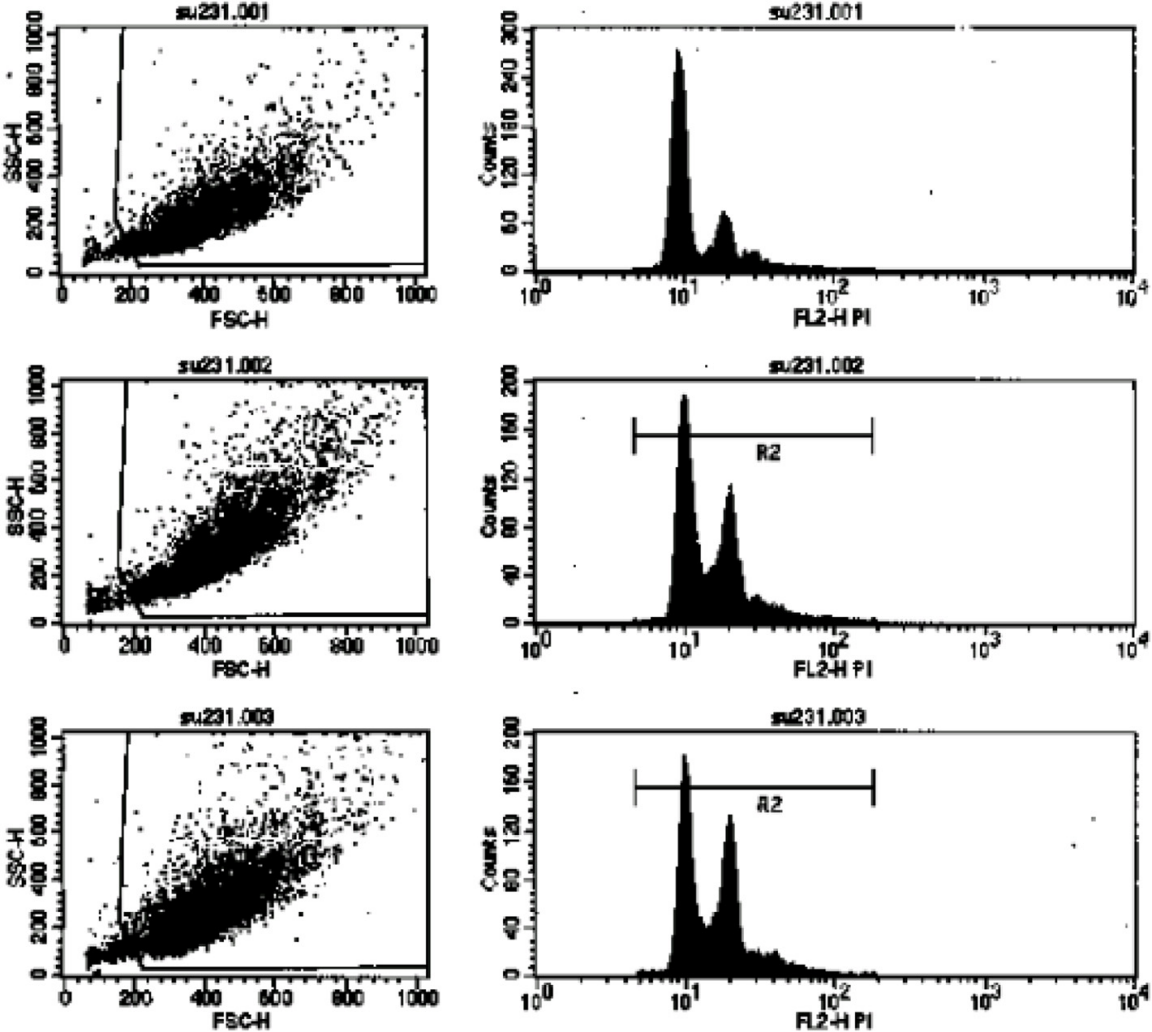
**DNA ploidy of MDA-MB-231 cells treated with SU6668.** 001: con group, 002: 15μM group, 003: 30μM group. MDA-MB-231 cells were cultured in media supplemented with SU6668(15μM, 30μM) for 72h, then incubated in PI solution for 30min, detected cell fluorescence intensity, which excitative wavelength was 488nm, and the emissive wavelength was 630nm. After SU6668 treatment, the tetraploid increased in a dose- dependent manner. With the increasing percentage of G2/M phase (tetraploid) cells, the percentage of G1 phase (diploid) cells was reduced.

After PI staining, SU6668 treated DNA content was tested by flow cytometry. The results showed that tetraploid increased after SU6668 processing in a dose dependence form. With the increasing percentage of G2/M phase (tetraploid) cells, the percentage of G1 phase (diploid) cells was reduced (Figure 4).

### SU6668 influenced MTDH distribution in MDA-MB-231 cells

**Figure 5:**
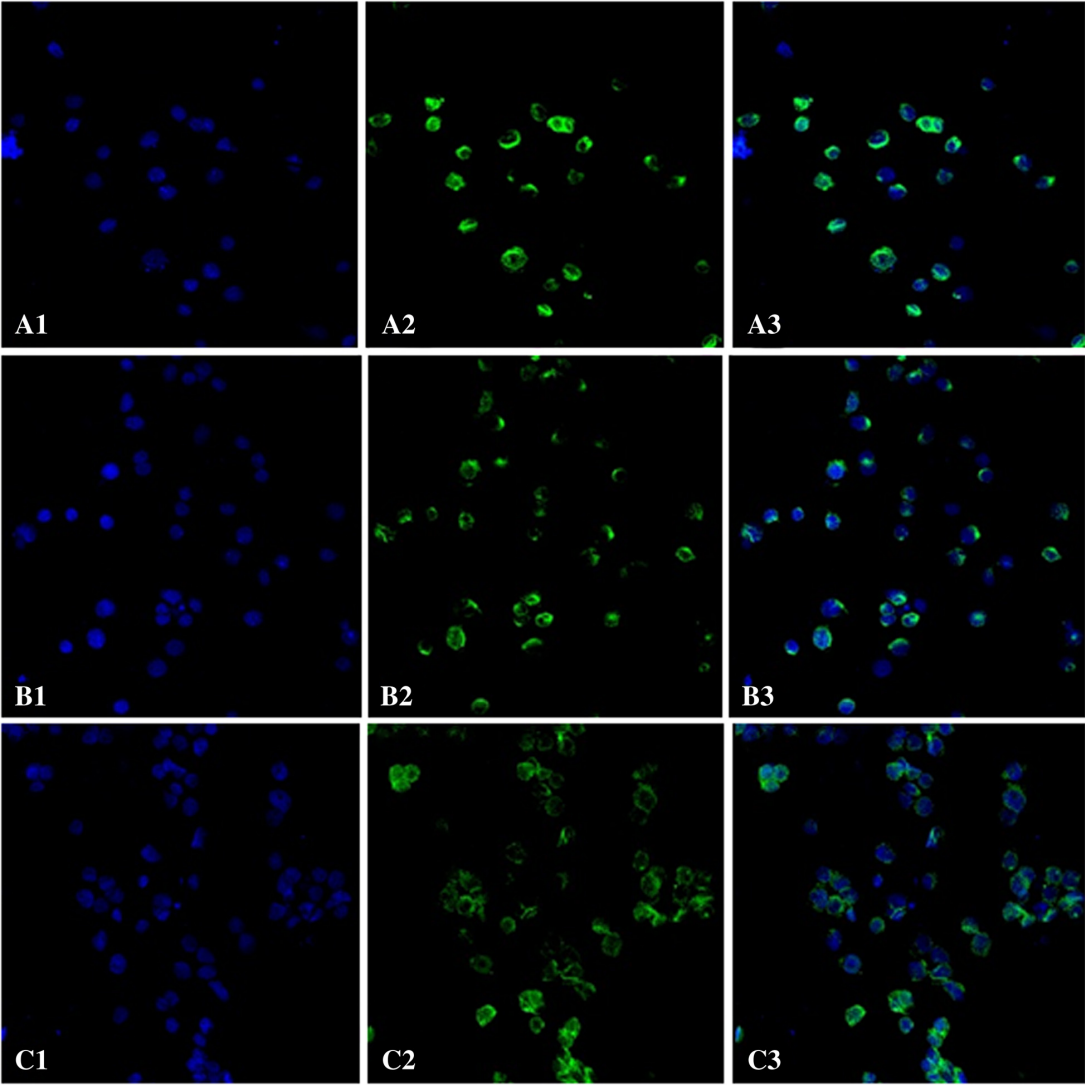
**MTDH distribution of MDA-MB-231 cells treated with SU6668. A**: con group; **B**: 15μM group; **C**: 30μM group (A1,B1,C1 showed nucleus tagged with blue fluorescent. A2, B2, C2 showed MTDH proteins tagged with green fluorescent. A3, B3, C3 were fused images). MDA-MB-231 cells were cultured in media supplemented with SU6668 (15μM, 30μM) for 72h.

Under laser confocal microscope, blue fluorescence represented the nuclei positive staining and green fluorescent represented MTDH protein positive staining. In the control group, green fluorescence was mainly distributed in the cytoplasm surrounding nucleus. In the treatment groups, the intensity of green fluorescence was decreased, less expressed in cytoplasm and relatively more expressed in nucleus. All of the findings were in a dose dependence form (Figure 5).

### SU6668 down-regulated VEGFR2, HIF-1α, MTDH, SMA and up-regulated E-cadherin in MDA-MB-231 cells

**Figure 6:**
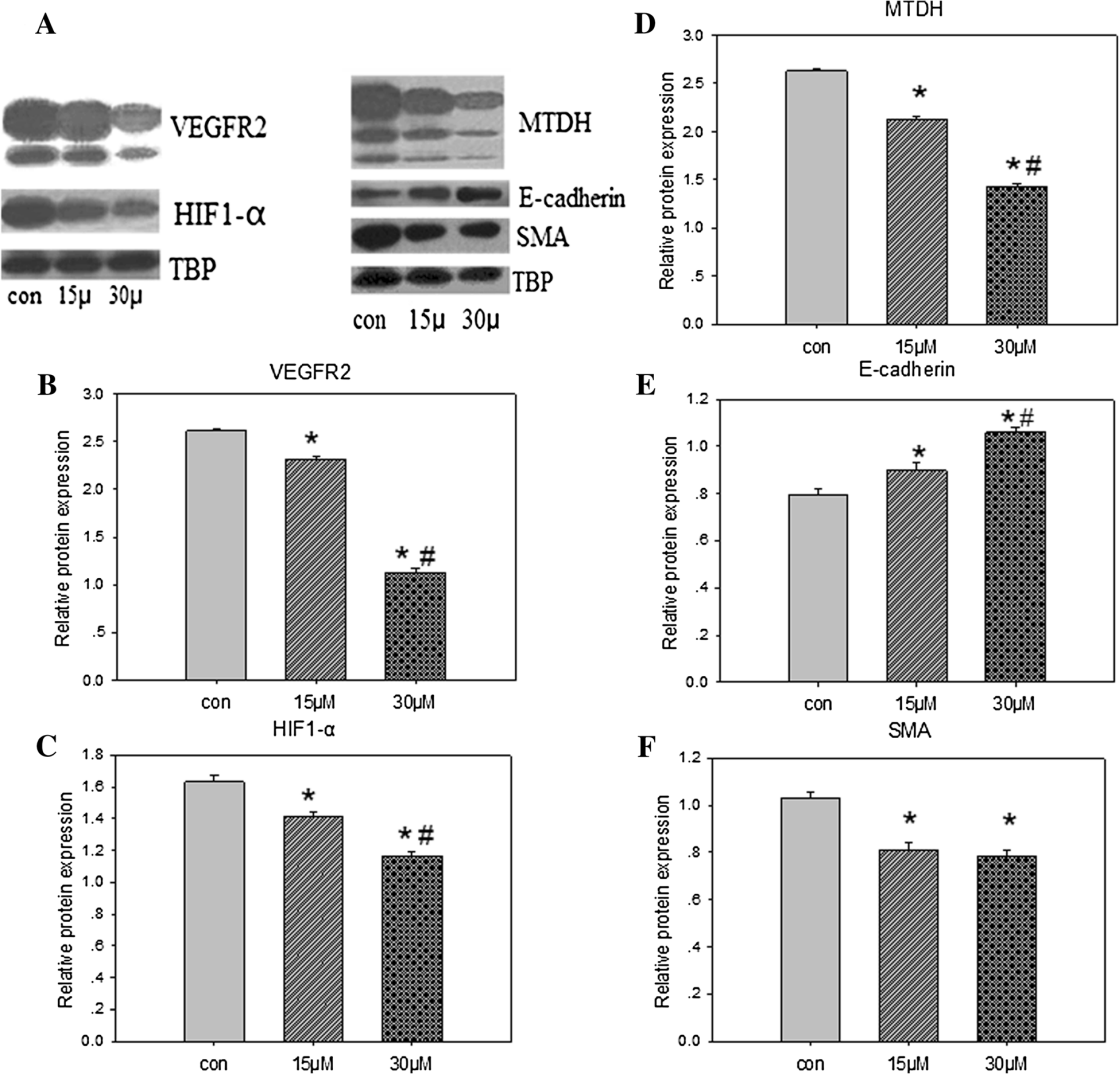
**Related proteins expression of MDA-MB-231 cells treated with SU6668.** Western blot showed that exposure to various concentrations of SU6668 (15μM, 30μM) for 72h resulted in related proteins expression change of MDA-MB-231 cells **(A)**. The protein expression of VEGFR2 **(B)**, HIF-1α **(C)**, MTDH **(D)**, SMA **(F)** were significantly decreased after SU6668 treatment, while the protein expression of E-cadherin **(E)** was increased obviously. The results were compared between each two groups. * *P* < 0.05, indicated significant differences from the control group, # *P* < 0.05, indicated significant differences from the 15μM group.

After dealing with SU6668, the expression of proteins related to angiogenesis, invasion and metastasis were detected by Western blotting. MDA-MB-231 cells were treated with 15 μM and 30 μM for 72 h, the expression of VEGFR2, HIF - 1α, MTDH, SMA protein were significantly decreased, while the expression of E-cadherin protein increased obviously (Figure 6).

## Discussion

TNBC is common in premenopausal young women, cell proliferation ratio is high, prognosis is poorer, the 5-year survival rate is less than 15% [13]. More and more small molecular compounds have been used to blocking the cell cycle, by inhibiting the mitosis process, promoting polyploidization and cell apoptosis, thus play a role of anti-tumor. Among them, the study from Gully and his colleague suggests a precursor of AZD1152 has potential therapeutic value for different molecular biology types of breast cancer, this finding provides a new choice that for TNBC treatment, which lacks of drug targets [7].

Scholars have devoted to tyrosine kinase inhibitor and have made great breakthroughs. Gleevec and Iressa have been approved by the United States FDA for separately used in treatment of chronic myelogenous leukemia and advanced non-small cell lung cancer, and acquire remarkable effect. SU6668, a kind of tyrosine kinase inhibitors, can act on VEGFR-2, PDGFR-beta and FGFR1, competing with ATP binding site so as to inhibit the activity of tyrosine kinase [9]. Its treatment decreased tumor cell proliferation, induced tumor endothelial cell apoptosis. Intraperitoneal injection or oral SU6668 could inhibit growth of transplantation tumor like glioma, melanoma, lung cancer, colon cancer, ovarian cancer in nude mouse [9].

Hu and his colleague found that MTDH gene was over-expressed in more than 40% of breast cancer patients, which not only promoted the metastasis of tumor cells, but also enhanced the resistance to chemotherapy drugs and affected the clinical therapeutic effect [14]. Recently, Li reported that MTDH was not expressed in normal mammary epithelial cells, but expressed with different extent in breast cancer cell lines [5]. The expression of MTDH gene positively correlated with the clinical stage of breast cancer, prompting that MTDH gene expression may be considered as an independent prognostic indicator for breast cancer [5]. Howerver, the function and significance of MTDH gene in TNBC cells have been rarely reported.

Epithelial-mesenchymal transformation (EMT) play an irreplaceable role in tumor proliferation and invasion process, and are also closely related with tumor distant metastasis and drug resistance [15]. Studies suggested that up-regulated MTDH expression in MCF-7 cells, protein fiber connection expression was increased, and E-cadherin expression was reduced, that could promote the EMT process to enhance cell invasion and migration ability [5].

E-cadherin belongs to the family of cell adhesion molecule protein, and is widely expressed in various organizations [16]. Previous studies indicated that there was a close relationship between tumor metastasis and EMT, absent or suppressed expression of E-cadherin may be a key link in starting invasion and metastasis process, down-regulating E-cadherin expression can invalidate the adhesion function between cells, and lead to separation of adjacent cells [17]. The development of breast cancer may be associated with abnormal function of E-cadherin, so it is suggested to be an important indicator to detect the development of breast cancer [18].

α-SMA participates in the form of microfilament structure in eukaryotic cell, and acts as a cytoskeleton protein [19]. Higher expression of α-SMA in the tissues indicates the existence of EMT [20,21]. α-SMA expression has a certain relationship with tumor angiogenesis in a majority of tumors, which mainly affects the generation of tumor blood vessels directly or indirectly by regulating the activity of cell proliferation and the expression of VEGF [22].

The combination of VEGF and its receptors has a strong effect in angiogenesis, increasing vascular permeability, providing matrix to establish a new capillary network, and promoting the growth of tumor cells. Studies found that the expression of MTDH in TNBC had a positive relationship with the level of VEGF1 and microvascular density [23].

In the development of solid tumor, angiogenesis is insufficient to meet the rapid growth of the tumor blood supply, thus leads to local hypoxia, and up-regulates the expression of HIF-1α. The downstream genes are involved in enhancing tumor invasion and metastasis ability, so as to improve its adaptability to relatively low oxygen environment. The increased expression of HIF-1α is very common in a variety of human tumors and some precancerous lesions, indicating that its expression may be an early event in tumor progression [24].

Research showed that SU6668 played an important role in inhibiting proliferation and angiogenesis of tumor cells by down-regulating the expression of VEGFR2 and HIF-1α, [10,11]. Hypoxia induced activation of MTDH through PI3K/AKT pathway. Stabilizing the feedback of HIF-1α and MTDH, and activating PI3K would form a positive feedback loop to enhance tumor cells’ survive and the progression [25]. At the same time, it was reported that MTDH could also regulate EMT at the level of transcription through NF-κB pathway [26].

## Conclusions

Our study investigated for the first time the effect of SU6668 on TNBC cells, in order to provide research basis for developing new strategy in TNBC treatment. We found that the phenomenon of polyploidization appeared among cells, prompted that SU6668 processing induced mitotic arrest. SU6668 could inhibit angiogenesis and EMT through inducing DNA polyploidization, so as to inhibit the growth and proliferation of TNBC cells. Our results also showed that SU6668 down-regulated the expression of MTDH and other metastasis-related proteins, up-regulated the expression of metastasis suppressor. The findings indicated that MTDH may play a key role in signaling pathways of angiogenesis, invasion and metastasis in TNBC. However, the development of TNBC involved in multiple genes and multiple steps, further exploration on the potential target genes should be proceeded to obtain promising regimens for TNBC.

## Methods

### Cell cultures

The human TNBC cell line (MDA-MB-231) were cultured in high-glucose Dulbecco’s Modified Eagle Medium(DMEM; Gibco Corporation, USA), supplemented with 10% fetal calf serum (Hyclone, USA) at a temperature of 37°C, 5% CO_2_, 95% oxygen, and 95% humidity.

### MTT

1 × 10^3^ MDA-MB-231 cells were planted into 96-well plates. After 24 h, different concentration of SU6668 was added to culture medium, and continued to cultivate for 72 h. MTT (20 μl/well) was added and developed for 4 h, DMSO (150 μl/well) was added finally. The absorbance value (A) was measured with a microplate reader. The concentration-suppression curve was drawn, and the inhibition rate was calculated. Inhibition rate = (1 - experimental group A/control group A) × 100%.

### Tablet cloning

200 MDA-MB-231 cells were planted into 9 cm petri dish. After 72 h SU6668 processing, PBS was used to wash cells for 3 times and fresh medium was added. After three weeks, 5 ml methanol was added to fix cells for 15 min. Giemsa was used to stain cells for 20 min, washed off with slow water, and was dried by air. The clone formation rate was calculated under microscope, clone formation rate (%) = (clone number/plated cell number) × 100%.

### Flow cytometric

Processed the logarithmic phase of MDA-MB-231 cells with SU6668 for 72 h, collected cells and fixed punch with cold methanol, washed with PBS for three times, then incubated in PI solution including RNaseA at room temperature away from light, after 30 min, detected cell fluorescence intensity by flow cytometry instrument, which excitative wavelength was 488 nm, and the emissive wavelength was 630 nm.

### Immunofluorescence

2.5 × 10^5^ MDA-MB-231 cells were planted into glass culture chambers. After 72 h SU6668 processing, washed with PBS for twice. 3% paraformaldehyde fixed at 4°C, 30 min. Washed with PBS for three times. Then, sections were incubated with first primary antibody MTDH (1:40) for 8 h at 4°C and Cy3-labeled goat anti-rabbit IgG (1:150) for 1 h at 25°C by turns. After being washed in PBS 3times, the nuclear antibody To-Pro3(1:1000)were added to the sections for 5 min at 25°C, then washed in PBS three times. The protein expression was observed by fluorescence microscope after being counterstained with DAPI and mounted with water-solubility mounting agents.

### Western bolt

Processed the logarithmic phase of MDA-MB-231 cells with SU6668 for 72 h, collected and cracked cells, extracted proteins, determined protein content with BCA method. Took 25 μg protein, separated with 12% sds-page method, transferred to the PVDF membrane, closed with 5% skim milk for 3 h at 25°C, added first primary antibody(1:100) for 8 h at 4°C, washed with TBST for three times, and second antibody (HRP-anti-rabbit 1:2,000, HRP-anti-Mouse 1:6,000, and HRP-anti-Biotin 1:6,000) for 2 h at 25°C, washed with TBST for three times. Took TBP as internal protein.

### Statistical analysis

Experiments were repeated for three times. Image J software was adopted to improve the half quantitative analysis of protein electrophoresis gray levels. The measurement data was expressed as mean ± standard deviation(SD). Statistical analysis, including ANOVA test, and q test, were carried out using the software package SPSS 16.0. The significance level was set at 5% for each analysis.

## Abbreviations

TNBC: Triple negative breast cancer; ER: Estrogen receptor; PR: Progesterone receptor; HER2: Human epidermal growth factor receptor type2; MTDH: Metadherin; AEG-1: Astrocyte elevated gene 1; EMT: Epithelial-mesenchymal transformation; SMA: Smooth muscle actin; PI: Propidium iodide; TBP: TATA-binding protein.

## Competing interests

The authors declare that they have no competing interests.

## Authors’ contributions

LW and ZZL carried out the molecular-biological studies and drafted the manuscript. DCM participated in its design helped to draft the manuscript.FG participated in the design of the study and performed the statistical analysis. XDX conceived of the study, and participated in its design and coordination. All authors have read and approved the final manuscript.
